# Less invasive skull base reconstruction using gelatin sponge and collagen matrix to prevent cerebrospinal fluid leakage after endoscopic transsphenoidal surgery: experience in 558 cases

**DOI:** 10.1007/s11102-025-01588-z

**Published:** 2025-10-22

**Authors:** Masanori Yonenaga, Shingo Fujio, Ryutaro Makino, Jun Sugata, Tomoko Hanada, Yushi Nagano, Nayuta Higa, Hitoshi Yamahata, Kazunori Arita, Koji Yoshimoto, Ryosuke Hanaya

**Affiliations:** 1 Department of Neurosurgery, Graduate School of Medical and Dental Sciences, Kagoshima University, 8-35-1 Sakuragaoka, Kagoshima, 890–8520 Japan; 2 Pituitary Disorders Center, Kagoshima University Hospital, Kagoshima, Japan; 3 Department of Neurosurgery, Izumi Regional Hospital, Akune, Japan; 4 Department of Neurosurgery, Graduate School of Medical Sciences, Kyushu University, Fukuoka, Japan

**Keywords:** Collagen matrix, CSF leakage, Endoscopic transsphenoidal surgery, Gelatin sponge, Skull base reconstruction

## Abstract

**Purpose:**

We evaluated the effectiveness of a less invasive skull base reconstruction technique using a fibrin glue-soaked gelatin sponge (FGGS) and collagen matrix to prevent cerebrospinal fluid (CSF) leakage following endoscopic transsphenoidal surgery (ETSS). This approach minimizes the need for fascia harvesting and the use of nasoseptal flaps.

**Methods:**

We retrospectively analyzed data from 558 ETSS, excluding Rathke’s cleft cysts, performed at our institution. Surgeries were classified by intraoperative CSF leakage grade according to Esposito’s classification. Patients’ characteristics and intraoperative findings were analyzed to assess potential risk factors for postoperative CSF leakage. Postoperative CSF leakage rates were compared between the pre-collagen matrix (428 surgeries) and collagen matrix (130 surgeries) periods.

**Results:**

FGGS-based reconstruction without fat grafts was performed in 73.7% of surgeries, including 99.4% of grade 0 and 88.5% of grade 1 leaks. Nasoseptal flaps were used in only eight surgeries (1.4%), and fascia was not used at all, either for watertight dural suturing or for multilayer reconstruction. The overall postoperative CSF leakage rate was 1.8% (10/558). The leakage rates by intraoperative CSF leak grade were 0%, 1.7%, 5.6%, and 1.1% for grades 0, 1, 2, and 3, respectively. Postoperative CSF leakage was associated with unfamiliar techniques and excessive bucking during extubation. During the collagen matrix period, although 33.8% of surgeries involved grade 3 high-flow CSF leaks, only one surgery (0.8%; 1/130) resulted in postoperative CSF leakage.

**Conclusions:**

As a less invasive technique, skull base reconstruction using FGGS and/or a collagen matrix was effective in reducing postoperative CSF leakage.

## Introduction

Over the last two decades, the indications for transsphenoidal surgery (TSS) have expanded with the advent of endoscopy. Endoscopic TSS (ETSS) has facilitated the resection of various skull base tumors, achieving higher rates of total gross resection with minimal invasiveness [1–3]. However, ETSS requires secure skull base reconstruction to prevent postoperative cerebrospinal fluid (CSF) leakage [4]. Various techniques and materials are used for the skull base reconstruction [5–7], with pedicle nasoseptal flap reconstruction widely recognized as a reliable method [8, 9]. However, it carries risks including septal perforation, prolonged crusting, cartilage necrosis, and olfactory loss [9–11]. Recently, the effectiveness of dural suturing or multilayer reconstruction with fascia has also been reported [12–15]. However, this technique is complex, challenging to master, and requires invasive fascia sampling.

Previously, we reported the effectiveness of fibrin glue-soaked gelatin sponge (FGGS) for skull base reconstruction. In this method, fibrinogen is absorbed into an absorbable gelatin-based sponge (Gelfoam^®^, Pfizer, New York, NY, USA), followed by the application of a 1:10 diluted thrombin solution before packing the tumor cavity. From April 2006 to September 2011, TSS was performed microscopically with FGGS applied in 255 cases for skull base reconstruction without pedicle nasoseptal flaps. Postoperative CSF leakage occurred in only 4 cases (1.6%) [16]. Since 2012, we have continued using FGGS for skull base reconstruction following the adoption of ETSS. However, owing to the supply uncertainty of Gelfoam since 2021, we introduced a collagen-based dural graft matrix (DuraGen^®^, Integra Lifesciences, Plainsboro, NJ, USA) as part of a new skull base reconstruction method, while also using alternative gelatin-based sponges. In this study, we describe our less invasive skull base reconstruction technique using FGGS, a collagen matrix, or a combination of both, and analyze its outcomes.

## Methods

### Patients

From August 2012 to December 2024, 621 ETSS procedures were performed at our institution. All surgeries were either directly performed or supervised by S.F., ensuring consistency in surgical technique and perioperative management. We excluded 43 surgeries for Rathke’s cleft cyst (RCC) because some RCC cases involved open drainage without skull base reconstruction. Furthermore, we excluded 11 surgeries in which neither FGGS nor a collagen matrix was used, as well as 9 surgeries in which the intraoperative CSF leakage grade could not be determined. Finally, we included 558 surgeries and retrospectively reviewed their intraoperative findings and postoperative outcomes.

### Intraoperative CSF leak grading

Intraoperative CSF leak was graded using the classification system proposed by Esposito et al. [17]: Grade 0, no detectable CSF leak on the Valsalva maneuver; Grade 1, very small CSF leak confirmed by the Valsalva maneuver, with or without a small diaphragmatic defect; Grade 2, moderate CSF leak confirmed with an obvious but not large diaphragmatic defect; and Grade 3, large CSF leak confirmed, with a significant diaphragmatic defect and exposure of nerves, vessels, and other subarachnoid space structures.

### FGGS Preparation

FGGS was prepared using an absorbable gelatin sponge (Gelfoam) and fibrin glue (Bolheal^®^, KM Biologics Co., Ltd., Kumamoto, Japan, or Beriplast^®^ P Combi-Set, CSL behring K.K., Tokyo, Japan). A 1 cm-wide Gelfoam strip was dipped in a fibrinogen solution, followed by immersion in a 1:10 diluted thrombin solution (1 mL thrombin mixed with 9 mL normal saline). Due to Gelfoam shortages, either Sponzel^®^ (LTL Pharma Co., Ltd., Tokyo, Japan) or Spongostan^®^ (Johnson & Johnson, New Brunswick, NJ, USA) was used as alternatives.

### Surgical procedure

**Fig. 1:**
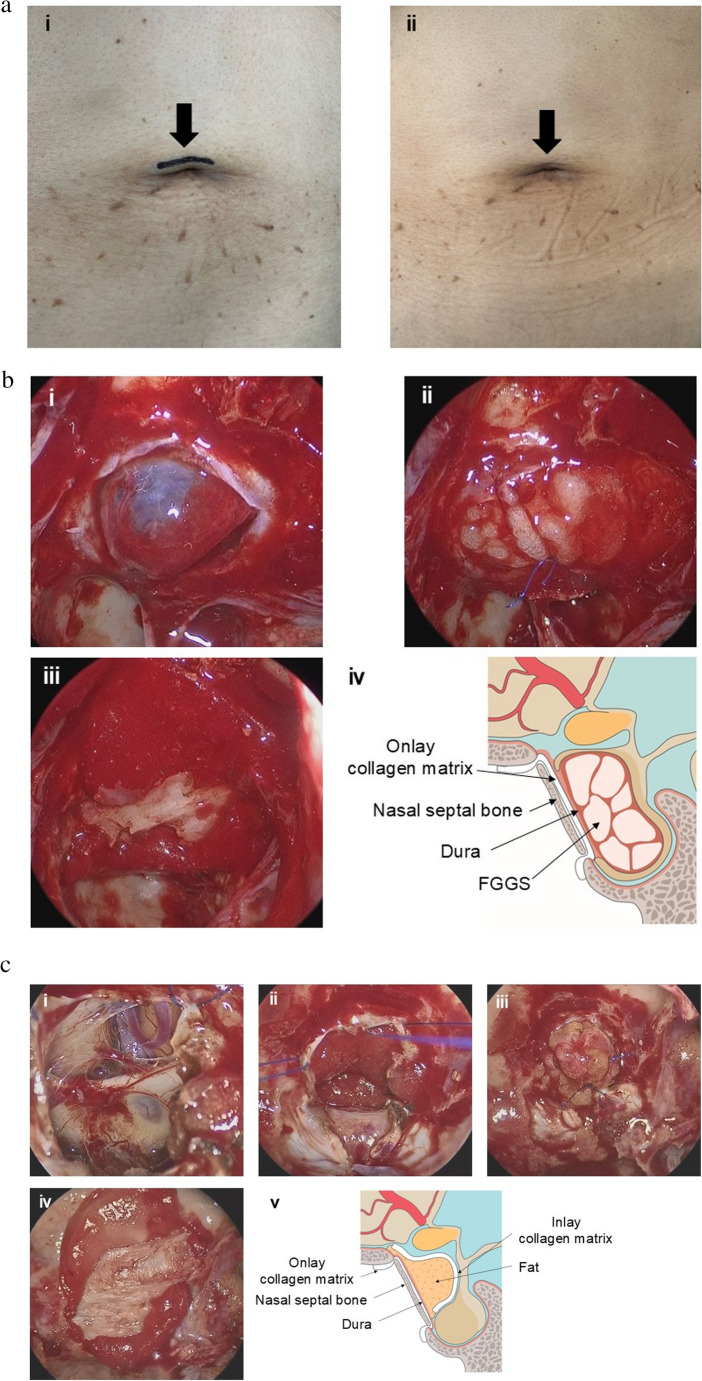
Fat harvesting technique and collagen matrix-assisted skull base reconstruction in Esposito’s grades 1 and 3. a. Fat harvesting from abdomen ⅰ: A 1–2-cm skin incision on the upper umbilical margin (arrow); ⅱ: One month after surgery. The skin incision is barely noticeable (arrow). b. The method of skull base reconstruction in Esposito’s grade 1 after introduction of collagen matrix (Case of nonfunctioning pituitary adenoma) ⅰ: After tumor removal, the arachnoid membrane has prolapsed. ⅱ: The tumor cavity is packed with multiple fibrin glue-soaked gelatin sponges (FGGSs), and the dura is sutured with one stitch. ⅲ: The skull base is covered with a collagen matrix, and a rigid reconstruction is created with the nasal septal bone. ⅳ: Schematic diagram of the method of skull base reconstruction in Esposito’s grade 1. c. The method of skull base reconstruction in Esposito’s grade 3 after introduction of collagen matrix (case of craniopharyngioma)

In 2012, we introduced a high-definition endoscope (Karl Storz SE & Co. KG, Tuttlingen, Germany) and fully transitioned to endoscopic endonasal surgery. As per our standard operative protocol, the nasal septal mucosa in the right nasal cavity was incised longitudinally at the anterior end of the middle turbinate. The contralateral nasal septal mucosa was incised approximately 2 cm anterior to this level to prevent postoperative septal perforation. After bilateral submucosal dissection, a piece of the nasal septal bone was harvested for rigid skull base reconstruction. The dura was incised in a cruciate fashion. Following tumor removal, skull base reconstruction was performed based on Esposito’s grading. For Esposito’s grade 0–1, the tumor cavity was packed with multiple FGGSs, and one or two stitches were placed in the dura using 6 − 0 Prolene^®^ (Ethicon, Johnson & Johnson, Somerville, NJ, USA) with a ValveGate^®^ needle holder (GEISTER, Tuttlingen, Germany). The nasal septal bone was then positioned at the base of the sella for rigid reconstruction and covered with FGGS. If the available septal bone was insufficient, artificial bone materials (hydroxyapatite ceramic: APACERAM^®^, or calcium phosphate bone paste: BIOPEX-R^®^, HOYA Technosurgical Co., Ltd., Tokyo, Japan) was used. Finally, fibrin glue was sprayed on the reconstructed surface. For Esposito’s grade 2–3, in which diaphragmatic defects increased the risk of postoperative CSF leakage, a 1–2 cm incision was usually made at the upper navel (Fig. 1a-ⅰ), and a small finger-sized fat graft was generally harvested and packed into the tumor cavity including the diaphragmatic defect area. As in Esposito’s grade 0–1, one or two stitches were placed in the dura, and rigid reconstruction was achieved using septal bone or artificial bone materials, with FGGS coverage. At the end of the procedure, fibrin glue was sprayed over the reconstructed skull base. The original reconstruction technique has been described in our previous publication [16], which served as the basis for the current procedure.

After tumor resection (ⅰ), the collagen matrix is inserted into the subdural space (ⅱ), and the tumor cavity is packed with abdominal fat. The dura is sutured (ⅲ), then the skull base is covered with a collagen matrix, and a rigid reconstruction is created with the nasal septal bone (ⅳ). ⅴ: Schematic diagram of the method of skull base reconstruction in Esposito’s grade 3.

The method of skull base reconstruction during the collagen matrix period was as follows. In Esposito’s grade 0–1 surgeries (Fig. 1b-ⅰ), the tumor cavity was packed with multiple FGGSs, and the dura mater was sutured with 1–2 stitches (Fig. 1b-ⅱ) and then covered with a collagen matrix, which was secured using the nasal septal bone or artificial bone materials (Fig. 1b-ⅲ). Subsequently, fibrin glue was sprayed. These techniques are illustrated schematically in Fig. 1b-ⅳ. In grade 2 surgeries, packing was predominantly performed with a fat graft, followed by dural suturing and collagen matrix coverage in the same manner. In grade 3 cases (Fig. 1c-ⅰ), a collagen matrix was first inserted into the subdural space (Fig. 1c-ⅱ), followed by fat graft placement between the incised dural edges. The dura was then sutured with 1–2 stitches (Fig. 1c-ⅲ), and a Valsalva maneuver (25 cmH₂O for 10 s) was performed to confirm the absence of CSF leakage. If a leakage was observed, additional dural sutures and fat packing were applied. The skull base was then covered with a collagen matrix, and rigid reconstruction was achieved (Fig. 1c-ⅳ). The Valsalva maneuver was repeated to confirm bone fixation. To finalize the reconstruction, fibrin glue was sprayed onto the surface. These techniques are illustrated schematically in Fig. 1c-ⅴ.

The detached bilateral nasal septal mucosa was repositioned to its original anatomical location, and nasal tampons (Merocel^®^, Medtronic, Minneapolis, MN, USA) were inserted into the bilateral nasal cavity to support and stabilize the repositioned mucosa. One tampon was removed on the second and the other on the third postoperative day.

The nasoseptal flap was used in very limited surgeries where skull base reconstruction was particularly challenging or when the internal carotid artery was exposed and required coverage. Lumbar drainage (LD) was selectively placed in patients at high risk of postoperative CSF leakage, such as those with craniopharyngioma and meningioma, and removed on the third postoperative day.

### Postoperative management

Postoperative computed tomography (CT) was routinely performed for all patients on the day of surgery to confirm the absence of complications such as hematoma or bone displacement. On the day of surgery, the patients remained on bed rest but were allowed to elevate their heads. On postoperative day 1, the patients were permitted to walk. Patients with LD remained on bed rest while the drain was in place, although head elevation was allowed with the drain clamped. Once the drain was removed, the bed rest restrictions were lifted.

### Statistical analysis

All statistical analyses were conducted using Starflex software (version 6.0; Artech Co. Ltd., Osaka, Japan) and OpenEpi (version 3.01; https://www.openepi.com/Menu/OE_Menu.htm). Based on the dataset characteristics, either the Mann–Whitney U test, chi-square test, or Fisher’s exact test was applied for data analysis. A p-value of < 0.05 was considered statistically significant.

## Results

### Patient characteristics and intraoperative CSF leakage rate

The study included 235 males and 284 female patients (median age: 58 [range: 8–88] years); 31 patients. underwent multiple surgeries. A total of 558 procedures were performed for the following disorders: 414 pituitary adenomas, 38 craniopharyngiomas, 20 meningiomas, 19 chordomas or chondrosarcomas, 14 liquorrhea (idiopathic or traumatic) or suspected liquorrhea, 8 metastatic tumors, 5 germinomas, 3 malignant lymphomas (MLs), 10 granulomas or hypophysitis, and 27 other conditions. The body mass index (BMI) at surgery was available for 530 procedures (median: 24.1 [range: 14.0–48.2] kg/m^2^). Sixteen surgeries were performed on patients with a history of radiation therapy (RT) to the head including the skull base. The intraoperative CSF leak grades were 0, 1, 2, and 3 in 181 (32.4%), 174 (31.2%), 108 (19.4%), and 95 (17.0%) surgeries, respectively.

### Skull base reconstruction techniques according to esposito’s grade

**Fig. 2:**
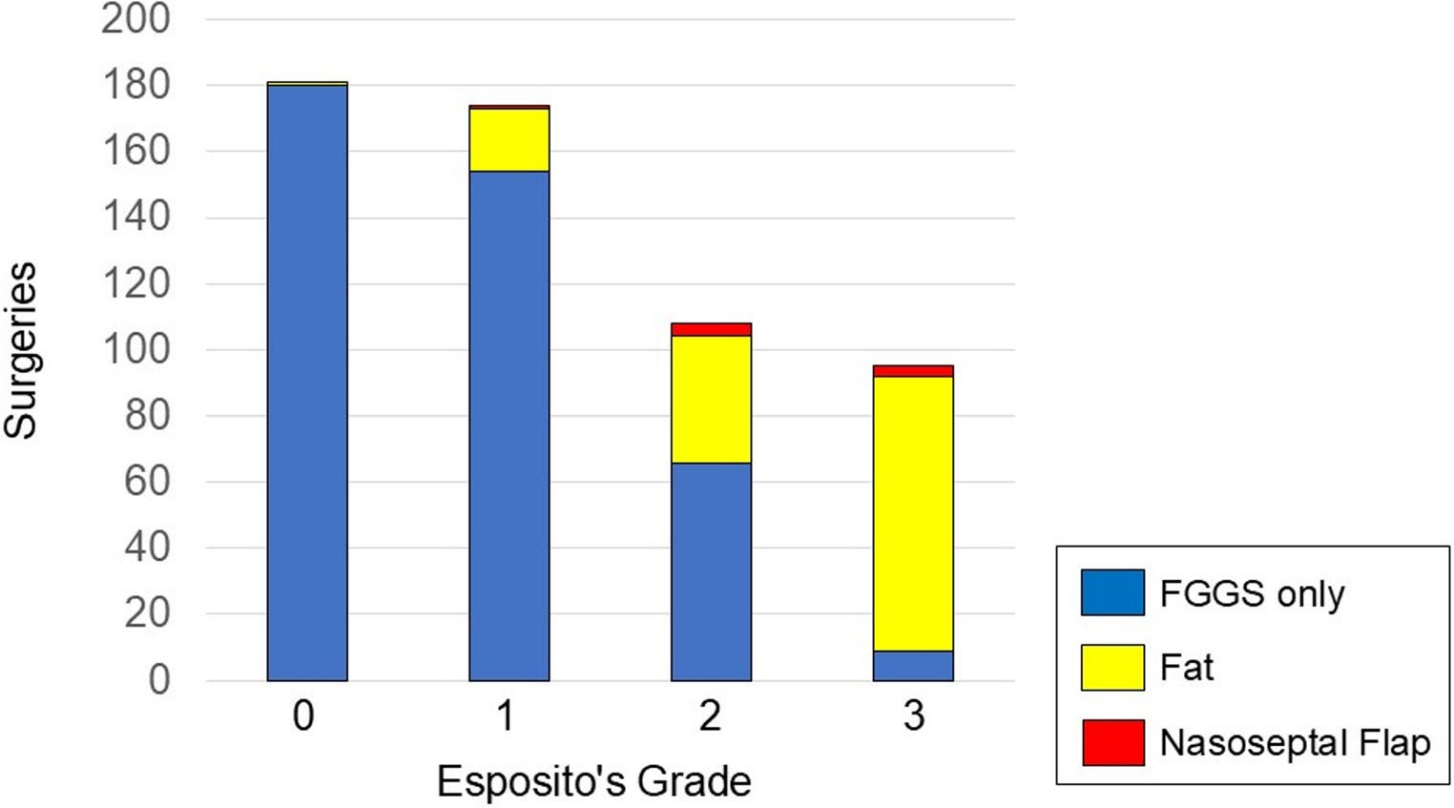
Skull base reconstruction techniques according to Esposito’s grade

Of the grade 0 surgeries, 180 (99.4%) were reconstructed with FGGS alone, and 1 (0.6%) with fat. Among the grade 1 surgeries, 154 (88.5%) were reconstructed with FGGS alone, 19 (10.9%) with fat, and 1 (0.6%) with a nasoseptal flap for idiopathic CSF leakage. Among the grade 2 surgeries, 66 (61.1%) were reconstructed with FGGS alone, 38 (35.2%) with fat, and 4 (3.7%) with a nasoseptal flap, including 1 case for coverage of an exposed internal carotid artery. For grade 3 surgeries, 9 (9.5%) were reconstructed with FGGS alone, 83 (87.3%) with fat, and 3 (3.2%) with a nasoseptal flap, 1 of which was for internal carotid artery coverage (Fig. 2). Fascia was not used in skull base reconstruction for any surgery.

The higher the Esposito grade, the greater the rate of fat used; however, the nasoseptal flap was rarely used.

### Frequency of LD and artificial bone materials

LD was used in 28 of 558 surgeries (5.0%): 1 (0.6%), 3 (2.8%), and 23 (24.2%) in Esposito’s grades 1, 2, and 3, respectively. LD was performed in one surgery (0.6%) of grade 0 for suspected but unconfirmed CSF leakage. Hydroxyapatite ceramic was used for rigid skull base reconstruction in 37 of 558 surgeries (6.6%): 6 (3.3%), 7 (4.0%), 11 (10.2%), and 13 (13.7%) of grades 0, 1, 2, and 3, respectively. Calcium phosphate bone paste was used instead of hydroxyapatite ceramic in 21 of 558 surgeries (3.8%): 3 (1.7%), 4 (2.3%), 2 (1.9%), and 12 (12.6%) of grades 0, 1, 2, and 3, respectively. Due to infection, calcium phosphate bone paste has not been used after 2018.

### Postoperative CSF leakage rate

There were 10 cases of postoperative CSF leakage (1.8%) (4 male and 6 female, median age: 60 [range: 15–75] years; Table 1). The postoperative CSF leakage rates according to the intraoperative CSF leak grade were 0% in grade 0, 1.7% (*n* = 3) in grade 1, 5.6% (*n* = 6) in grade 2, and 1.1% (*n* = 1) in grade 3. All cases were first-time surgeries with no history of RT. Compared with cases without CSF leakage, there was no significant difference in sex ratio (*p* = 0.93, Fisher’s exact test, odds ratio [OR]: 1.30, 95% confidence interval [CI]: 0.36–4.68), age (*p* = 0.99, Mann–Whitney U test), or BMI (*p* = 0.79, Mann–Whitney U test). One postoperative CSF leakage occurred in a case with artificial bone material use (Case 1), with no significant difference compared to non-use (*p* = 1.0, Fisher’s exact test, OR: 0.96, 95% CI: 0.15–6.27). Two cases involved unfamiliar techniques; the first instance of skull base reconstruction without abdominal fat for a grade 3 CSF leak of meningioma (Case 1) and the first use of a nasoseptal flap (Case 2). One case was of ML with delayed CSF leakage following tumor shrinkage after chemotherapy (Case 7). Another case resulted from intraoperative oversight of a small dural defect on the tuberculum sellae (Case 4). Two cases were associated with severe bucking during extubation (Cases 9 and 10). In all 10 cases, CSF leakage was resolved with a single reoperation.

**Table 1:** Detailed data on cases of postoperative cerebrospinal fluid leakage

Case	Age (Years)	Sex	Pathology	BMI (kg/m^2^)	Period	Previous surgery/RT	Intraoperative CSF leak grade	Adding fat/flap	Using artificial bone materials	CSF leakage sign	Duration to appearance of CSF leakage (Days)	Duration to reoperation (Days)	Presumed cause of CSF leakage	Reconstruction
1	45	Female	TSM	20.0	Pre-collagen matrix	-	3	－/－	＋	-	12	13	First case of FGGS-only use in grade 3 CSF leak of meningioma	Fat་Lumbar drainage
2	49	Female	GH-PA	21.6	Pre-collagen matrix	-	2	＋/＋	-	-	2	3	First case of flap use	Fat
3	64	Female	NF-PA	25.4	Pre-collagen matrix	-	1	－/－	-	-	5	7	Unknown	Fat
4	75	Female	NF-PA	24.1	Pre-collagen matrix	-	1	－/－	-	±	6	8	CSF leakage from a dural defect in tuberculum sellae	Fat
5	61	Male	NF-PA	24.3	Pre-collagen matrix	-	1	－/－	-	±	26	26	Unknown	Fat
6	59	Male	ACTH-PA	19.8	Pre-collagen matrix	-	2	－/－	-	-	22	22	Unknown	Fat
7	69	Male	ML	23.2	Pre-collagen matrix	-	2	－/－	-	-	45	46	Tumor shrinkage after HD-MTX	Fat
8	62	Female	NF-PA	26.0	Pre-collagen matrix	-	2	－/－	-	＋	2	7	Unknown	Fat
9	15	Female	ACTH-PA	33.0	Pre-collagen matrix	-	2	＋/－	-	＋	1	2	Bucking during extubation	Fat
10	57	Male	NF-PA	25.3	Collagen matrix	-	2	－/－	-	＋	5	6	Bucking during extubation	Fat

ACTH-PA; adrenocorticotropic hormone–producing pituitary adenoma, BMI, body mass index; CSF, cerebrospinal fluid; FGGS, fibrin glue-soaked gelatin sponge; GH-PA, growth hormone–producing pituitary adenoma; HD-MTX, high-dose methotrexate; ML, malignant lymphoma; NF-PA, nonfunctioning pituitary adenoma; RT, radiation therapy; TSM, Tuberculum sella meningioma

### Comparison of pre-collagen matrix period and collagen matrix period

We investigated the impact of a collagen matrix on skull base reconstruction in ETSS. The baseline characteristics and postoperative CSF leakage rates between the pre-collagen (*n* = 428) and collagen matrix (*n* = 130) periods are shown in Table 2. The collagen matrix period included a significantly higher proportion of craniopharyngioma cases (24/130, 18.5% vs. 14/428, 3.3%; *p* < 0.01, chi-square test, OR: 6.70, 95% CI: 3.35–13.39) and Esposito’s grade 3 CSF leaks (44/130, 33.8% vs. 51/428, 11.9%; *p* < 0.01, chi-square test, OR: 3.78, 95% CI: 2.37–6.03). Despite these risk factors, postoperative CSF leakage occurred in only one case during the collagen matrix period (1/130, 0.8%), compared to nine cases in the pre-collagen matrix period (9/428, 2.1%), although this difference was not statistically significant (*p* = 0.47, Fisher’s exact test; OR: 0.36, 95% CI: 0.045–2.88). LD was used more frequently during the collagen matrix period (11/130, 8.5% vs. 17/428, 4.0%; *p* = 0.04, chi-square test, OR: 2.24, 95% CI: 1.01–4.97), because of initial concerns regarding the new technique. Among 106 surgeries performed since May 2022, when we became accustomed to the use of collagen matrix, only 2 (1.9%) required LD.

**Table 2:** Comparison of baseline characteristics and postoperative CSF leakage rates between the pre-collagen and collagen matrix periods

	Pre-collagen matrix period	Collagen matrix period	*p*-value
Total Surgeries	428	130	n/a
Median BMI (range), kg/m^2^	24.1^a^ (14.0–48.2)	24.3^b^ (16.6–41.4)	0.88^c^
Primary ETSS/Repeated ETSS	357/71	113/17	0.34^d^
Previous RT	13	3	0.77^e^
Disorders			
Pituitary adenoma	332	82	< 0.01^d^
Craniopharyngioma	14	24	< 0.01^d^
Meningioma	12	8	0.07^d^
Chordoma/Chondrosarcoma	16	3	0.59^e^
Others	54	13	0.42^d^
Esposito’s Grade (0–2/3)	377/51	86/44	< 0.01^d^
Use of lumbar drainage	17	11	0.04^d^
Use of artificial bone materials	41	17	0.25^d^
Postoperative CSF leakage	9 (2.1%)	1 (0.8%)	0.47^e^

Some data were missing for some variables. a, *n* = 402; b, *n* = 128

c, Mann–Whitney U test; d, Chi-square test; e, Fisher’s exact test

BMI, body mass index; CSF, cerebrospinal fluid; ETSS, endoscopic transsphenoidal surgery; FGGS, fibrin glue-soaked gelatin sponge; n/a, not applicable; RT, radiation therapy

### Complications

Three patients developed postoperative meningitis: one in the pre-collagen matrix period (with LD) and two in the collagen matrix period (without LD). None exhibited obvious CSF leakage, and all patients responded to antibiotic therapy. One patient in the collagen matrix period later developed hydrocephalus requiring shunting. A patient with a history of TSS developed a postoperative pituitary abscess in the pre-collagen matrix period, which was resolved with drainage and antibiotics. In addition, two cases of infection associated with calcium phosphate bone paste occurred in the pre-collagen matrix period. Another patient with LD in the collagen matrix period developed a chronic subdural hematoma, requiring drainage. One patient required re-stitching due to wound dehiscence at the abdominal fat extraction site.

### Representative cases (Case 10)

**Fig. 3:**
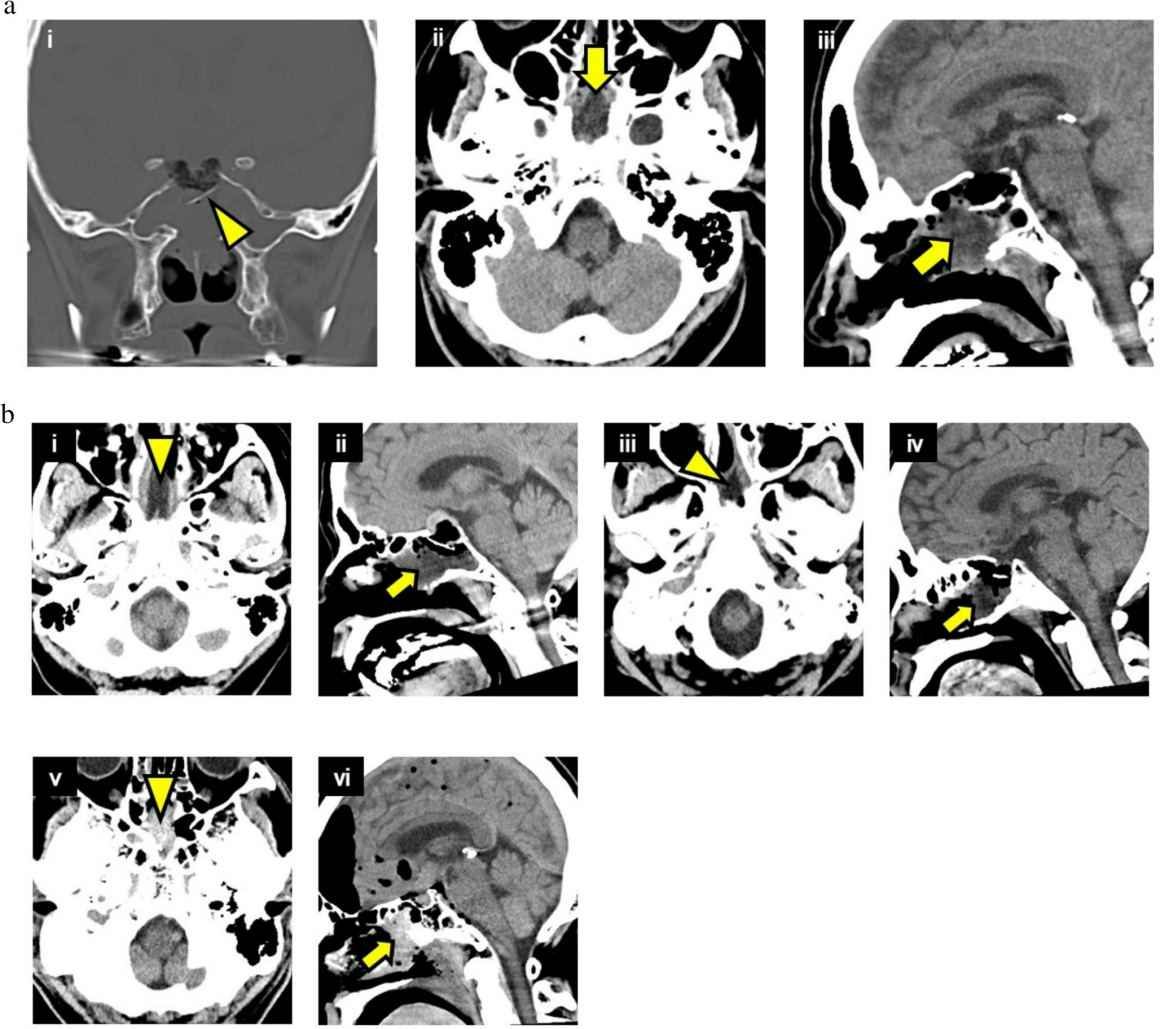
Postoperative computed tomography (CT) findings in Case 10 and early cerebrospinal fluid leakage sign a: Case 10: A 57-year old man. Postoperative CT revealed prolapse of the nasal septal bone (arrowhead in ⅰ) and cerebrospinal fluid retention under the nasal mucosa and within the sphenoid sinus (arrows in ⅱ and ⅲ). b: Early cerebrospinal fluid leakage sign

A 57-year-old Japanese man with a history of smoking had a nonfunctioning pituitary adenoma, presenting with visual impairment. Gross-total resection of the tumor was achieved. The suprasellar arachnoid membrane was partially damaged, and an Esposito’s grade 2 CSF leakage was observed intraoperatively. FGGS was packed into the tumor cavity, successfully stopping CSF outflow. Additionally, a collagen matrix was applied to cover the surface, and sella-floor plasty was performed using the patient’s nasal septal bone. To ensure repair integrity, airway pressure was elevated to 25 cmH₂O via the Valsalva maneuver, confirming the absence of further CSF leakage. However, the patient experienced severe bucking during extubation. Postoperative CT revealed nasal septal bone prolapse, along with CSF retention under the nasal mucosa and within the sphenoid sinus (Fig. 3a-ⅰ–ⅲ). One nasal pack was removed on postoperative day 2 and the other on postoperative day 3. On postoperative day 5, CSF leakage was observed, necessitating surgical revision on postoperative day 6. Intraoperatively, deviation of the nasal septal bone used for sella-floor plasty was identified. The repair was reinforced using autologous fat grafts, and no CSF leakage recurrence was observed after the second operation.

Cases 8 (ⅰ, ⅱ) and 9 (ⅲ, ⅳ) show signs of submucosal nasal (arrowhead) or intrasphenoidal sinus (arrow) cerebrospinal fluid (CSF)-like fluid accumulation on CT immediately after surgery. In contrast, Case 7 (ⅴ, ⅵ), with delayed CSF leakage due to tumor shrinkage from chemotherapy, has a hematoma, not CSF, in the submucosal nasal (arrowhead) and sphenoid sinus (arrow).

### Early cerebrospinal fluid leakage sign

Case 10 was considered to have symptomatic CSF leakage after tampon removal, as the bilateral nasal septal mucosa was compressed by the nasal packing, despite the CSF leakage appearing at extubation due to bucking. Thus, the presence of submucosal nasal or intrasphenoidal sinus CSF-like accumulation on immediate postoperative CT may indicate an early CSF leakage. Similarly, in Cases 8 and 9, in which CSF leakage symptoms appeared earlier, CSF accumulation was suspected under the nasal mucosa and within the sphenoid sinus on immediate postoperative CT (Fig. 3b-ⅰ–ⅳ). In contrast, Case 7, which developed delayed CSF leakage following tumor shrinkage due to chemotherapy, did not exhibit these finding (Fig. 3b-ⅴ,ⅵ).

## Discussion

Our less invasive skull base reconstruction technique, performed without a nasoseptal flap or fascia-based watertight dural suturing or multilayer reconstruction, was associated with a CSF leakage rate of 1.8%, supporting its feasibility as a reconstruction approach. Notably, the CSF leakage rate was 0.8% in the 130 cases where a collagen matrix was introduced.

As ETSS indications expand, the risk of postoperative CSF leakage also increases due to the need for more complex skull base reconstruction, with reported CSF leakage rates following ETSS of 1.2–16.7% [5, 13–15, 18–30]. Among various risk factors, high-flow CSF leakage in Esposito’s grade 2–3 cases is a significant contributor [21, 26, 30]. Kuan et al. reported that among 300 cases, postoperative CSF leakage incidence was 1.0% in Esposito’s grade 0, 2.2% in grade 1, 9.4% in grade 2, and 6.7% in grade 3, highlighting the increased risk in higher-grade leaks [25]. Accordingly, as the Esposito’s grade increases, more robust skull base reconstruction is required. Hara et al. described that in grade 1–2 CSF leaks, FGGS seals the leakage points, with abdominal fat grafts filling the sellar space and repairing the dural defects. For grade 3 leaks, a fascial patch graft from the right thigh is secured with 6–8 sutures as an inlay graft, reinforced with a pedicled nasoseptal flap [30]. Ishikawa et al. reported that in grade 2 cases, abdominal fat fills the defect, followed by simple dural suturing. In grade 3 cases without considerable dural defect, continuous dural suturing with fat is followed by a rigid reconstruction. When grade 3 cases involve large dural defects, continuous dural suturing with the fascia replaces fat [15]. In line with these findings, nasoseptal flaps and fascia graft dural suturing have been highly effective for high-flow CSF leakage [14, 20, 26, 28]. However, nasoseptal flap reconstruction carries risks such as septal perforation, prolonged mucosal crusting, cartilage necrosis, and olfactory dysfunction [9–11]. Moreover, fascia lata grafts from the thigh or abdomen may lead to postoperative donor site complications, including pain, wound discharge, and hematoma [31]. Furthermore, dural suturing is time-consuming; although the duration shortened with experience, the mean time for dural suturing remained at 85.8 ± 22.5 min [28]. In our practice, we have limited the procedure to placing only one or two stitches to approximate the dura, which requires about 5–10 min. In Esposito’s grades 0–1, where FGGS is mainly used for skull base reconstruction, the contribution of this limited suturing to the prevention of CSF leakage remains uncertain. However, in grade 2–3 cases requiring fat grafting, we believe that even this limited suturing is helpful because it secures the fat between the dural edges, thereby providing additional stability. Thus, our less invasive technique, avoiding the harvesting of fascia or nasoseptal flaps, offers a simple and effective approach with a low postoperative CSF leakage rate.

Abdominal fat grafts are widely used in skull base reconstruction, particularly for CSF leak repairs and filling dead space after tumor removal [21, 32, 33]. In this study, fat grafts were utilized in 147 surgeries (26.3%), primarily for grades 2–3 CSF leaks. Some reports have described the use of fat grafts as a packing material, even in grade 0–1 CSF leaks, which exhibit minimal or no intraoperative leakage [26, 32]. We consider FGGS an effective alternative, as it fills the dead space while preventing minor CSF leakage, making it sufficient for grade 0–1 cases. Although harvesting abdominal fat requires an additional incision, we only used a small intraumbilical incision, which is cosmetically advantageous (Fig. 1a-ⅱ) [33].

Previously identified risk factors for postoperative CSF leakage following ETSS include a history of repeated surgeries [21], high BMI [20, 23], prior RT [20], and tumor type, particularly craniopharyngioma and chordoma [20, 30]. However, we found no significant association between these factors and postoperative CSF leakage occurrence. Several CSF leakage cases in our series were associated with the use of an unfamiliar technique, reflecting the influence of surgical learning curve. The learning curve in ETSS is well recognized, with CSF leak rates decreasing as surgeons gain experience [26, 32]. Considering this, comprehensive operator training and gradual familiarization with new techniques are crucial to minimizing postoperative CSF leakage.

We compared the frequency of postoperative CSF leakage before and after the introduction of the collagen matrix. Despite a high proportion of Esposito’s grade 3 high-flow CSF leaks, the frequency of postoperative CSF leakage remained low (0.8%) during the collagen matrix period. Since its introduction in Japan in 2019, the collagen matrix has been adopted as an optional material for dural reconstruction in neurosurgical procedures. Its porous structure supports fibroblast infiltration, promoting the development of a neomembrane [29, 34]. Building on its successful application in numerous craniotomies, the collagen matrix has recently been used for skull base reconstruction in ETSS [27, 29]. However, Nagata et al. reported that postoperative CSF leakage was observed at the junction between the coagulated dura and adjacent collagen matrix, indicating that sufficient intact adjacent dura is crucial for proper neovascularization and neomembrane formation [29]. Compared to that in craniotomy, the area of the dura mater available for collagen matrix adhesion is limited in ETSS. To address this limitation, rigid reconstruction may help promote more stable adhesion of the collagen matrix to the dura mater. In Case 10, postoperative CSF leakage occurred following the immediate failure of rigid reconstruction, suggesting a potential association. However, the overall effectiveness of rigid reconstruction in preventing CSF leakage remains unclear [20]. Further research is necessary to clarify the clinical utility of combining the collagen matrix with rigid reconstruction.

Regarding safety, although infrequent, several postoperative events were observed. Among them, two cases of meningitis occurred during the collagen matrix period, even though LD was not used; therefore, a potential influence of the collagen matrix cannot be completely excluded. While these findings do not raise new safety concerns specific to the collagen matrix, larger cohorts and longer follow-up will be needed to further establish its long-term safety profile.

LD can be beneficial in reducing the risk of CSF leakage in ETSS [22, 24]. By maintaining postoperative drainage, intracranial pressure is lowered, providing an alternative pathway for CSF egress. This process supports dural defect healing and may aid in managing high-flow CSF leaks. However, there are potential risks associated with LD, such as spinal headaches, infection, catheter retention, and overdrainage [18, 22, 24]. We used LD in 5% of our procedures, mainly for high-flow CSF leaks. Complications occurred in two cases: one infection and one chronic subdural hematoma. During the collagen matrix period, LD was used more frequently in the early phase due to limited experience with the new reconstruction technique. As the procedure became more established, LD use gradually declined. Nevertheless, LD may remain valuable in selected high-risk cases, particularly Esposito’s grade 3 leaks. Therefore, whether its use can be avoided once the reconstruction technique is established remains debatable.

We believe that postoperative CSF leakage occurs in two distinct patterns. The first pattern is immediate CSF leakage, which occurs shortly after the surgery. In Cases 9 and 10, severe bucking occurred during extubation, and CT performed immediately after surgery showed early CSF leakage signs within the sphenoid sinus and bilateral subnasal mucosa. Although nasal packing in the bilateral nasal cavities may delay the onset of symptoms for several days postoperatively, CSF leakage was likely present immediately after surgery in these cases. Bucking and vomiting before and after extubation are recognized risk factors for postoperative CSF leakage [26]. Therefore, close coordination with anesthesiology to prevent bucking and vomiting is crucial, and careful follow-up is warranted when early CSF leakage signs are observed. The second pattern is delayed CSF leakage, which may develop over time as the tumor shrinks with treatment, as demonstrated in Case 7. Similarly, even when skull base reconstruction is performed using FGGS alone, the possibility of delayed CSF leakage must be considered. Since gelatin sponges begin to be absorbed in approximately 2 weeks [35], the reconstructed skull base is expected to heal naturally as the gelatin-based materials gradually dissolve. However, if resorption occurs before sufficient tissue integration, CSF leakage may develop. This was observed in Case 1, in which FGGS alone was used for a Esposito’s grade 3 intraoperative CSF leak, resulting in CSF leakage on postoperative day 12. Therefore, in cases of grade 2–3 high-flow CSF leakage, it is advisable to reinforce the repair by adding abdominal fat.

This study has some limitations. First, our experience with more extensive endoscopic skull base surgeries, such as the endoscopic transsphenoidal transclival approach, was limited. A larger number of cases is required to evaluate the effectiveness of our technique for these more invasive procedures. Second, the lower postoperative CSF leakage rate observed during the collagen matrix period might, at least partly, reflect the cumulative increase in surgical experience and procedural refinement over time. The more frequent use of LD in the early phase of this period might also have contributed. Third, the number of postoperative CSF leakage cases in the collagen matrix period was very small, and the imbalance in cohort size between the pre-collagen matrix (428 surgeries) and collagen matrix (130 surgeries) period reduced the statistical power to detect significant differences. Therefore, the independent efficacy of the collagen matrix and the precise risk factors for postoperative CSF leakage should be interpreted with caution. Fourth, the discontinuation of Gelfoam necessitated the use of alternative gelatin-based products, such as Sponzel and Spongostan. In the 130 surgeries where a collagen matrix was introduced—most of which used FGGS prepared with these substitutes—postoperative CSF leakage remained rare. However, this heterogeneity in reconstruction materials may have introduced some bias when evaluating the efficacy of the collagen matrix.

In conclusion, our less invasive skull base reconstruction technique, in which FGGS and/or a collagen matrix are used, effectively prevents postoperative CSF leakage in ETSS without the use of nasoseptal flaps or fascia-based watertight dural suturing and multilayered reconstruction techniques. The introduction of a collagen matrix was associated with further reduction in leakage rates, suggesting a possible role as an alternative material for skull base reconstruction after ETSS.

## Data Availability

The data that support the findings of this study are not publicly available due to patient confidentiality and ethical restrictions, but are available from the corresponding author upon reasonable request.
